# The Cut-Off Value for the Triglyceride–Glucose Index (TyG) Discriminating Insulin Resistance Based on the SHBG Level and HOMA-IR Values in Women with Polycystic Ovary Syndrome

**DOI:** 10.3390/life15050691

**Published:** 2025-04-23

**Authors:** Tahar Ben Rhaiem, Aleksander Jerzy Owczarek, Mariusz Wójtowicz, Dariusz Ciura, Paweł Madej, Jerzy Chudek, Magdalena Olszanecka-Glinianowicz

**Affiliations:** 1 Department of Gynecological Endocrinology, Faculty of Medical Sciences in Katowice, Medical University of Silesia in Katowice, 40-752 Katowice, Poland; benrhaiem66@interia.eu (T.B.R.); pmadej@interia.pl (P.M.); 2 Health Promotion and Obesity Management Unit, Department of Pathophysiology, Faculty of Medical Sciences in Katowice, Medical University of Silesia in Katowice, 40-752 Katowice, Poland; aowczarek@sum.edu.pl (A.J.O.);; 3 Clinical Department of Gynecology and Obstetrics, Faculty of Medical Sciences in Zabrze, Medical University of Silesia in Katowice, 41-803 Zabrze, Poland; mariuszwojtowicz007@onet.eu; 4 Department of Internal Medicine and Oncological Chemotherapy, Faculty of Medical Sciences in Katowice, Medical University of Silesia in Katowice, 40-029 Katowice, Poland; chj@poczta.fm

**Keywords:** insulin resistance, PCOS, HOMA-IR, SHBG, TyG

## Abstract

The triglyceride–glucose (TyG) index was recently suggested as a surrogate marker of liver steatosis and insulin resistance. However, the TyG index cut-off value may be affected by age, sex, and race. Therefore, this study aimed to estimate the cut-off value for the TyG index discriminating insulin resistance based on the previously established cut-offs for HOMA-IR (Homeostatic Model Assessment for Insulin Resistance) and serum level of SHBG (sex hormone-binding globulin) in Caucasian women with polycystic ovary syndrome (PCOS). The medical records of 311 unselected Caucasian women diagnosed with PCOS were included. Finally, due to the exclusion of patients with diabetes and hypertension, a cohort of 264 (84.9%) women with PCOS were analyzed. The following data were retrieved from the medical history: age, body weight, waist circumference, height, serum levels of glucose, triglycerides, insulin, and SHBG. HOMA-IR was calculated with a standard formula. The TyG index was calculated according to the formula TyG = ln(triglycerides[mg/dL] × glucose [mg/dL]/2). The cut-off value for the TyG index was calculated using ROC analysis. The empirical optimal of the TyG index cut-off, corresponding to HOMA-IR ≥ 2.1, was >8.31 (AUC 0.77, accuracy 0.70, sensitivity 61.2%, specificity 75.3%, PPV—positive predictive value 59.4%, NPV—negative predictive value 76.7%). The corresponding TyG index cut-off values for a SHBG level < 41.5 nmol/L was >8.31 (AUC 0.67, accuracy 0.65, sensitivity 54.9%, specificity 73.9%, PPV 64.4%, NPV 65.6%). Our study suggests that the cut-off point for the TyG index in young Caucasian women with PCOS, which discriminates against insulin resistance, is 8.31 (based on both HOMA-IR and SHBG values). In addition, our data confirm the usefulness of the TyG index as an initial assessment of insulin resistance, which should be confirmed by assessing the HOMA-IR value or SHBG concentration.

## 1. Introduction

The role of insulin resistance and compensatory hyperinsulinemia in the development of hyperandrogenism has been well established. Increased insulin levels stimulate testosterone synthesis in the thecal ovary cells and the reticular layer of the adrenal cortex [1,2]. Insulin resistance also plays a key role in the development of metabolic disorders that develop in women with PCOS, such as carbohydrate metabolism disturbances and type 2 diabetes, as well as atherogenic dyslipidemia, which results in increased cardiovascular risk [3,4,5,6,7]. In turn, the mitogenic effect of excess insulin is one of the main risk factors for the development of endometrial and breast cancer [8,9].

The gold standard for assessing insulin resistance is the hyperinsulinemic–euglycemic clamp (HIEC). This method especially assesses muscle insulin resistance [10]. It is rarely used in scientific study and clinical practice as it is time consuming, burdensome for the examined person, and expensive. In addition, there is no widely accepted cut-off point for the glucose infusion rate when the hyperinsulinemic steady-state is achieved. The mathematically developed Homeostatic Model Assessment Insulin Resistance (HOMA-IR) is the most commonly used method for assessing insulin resistance, based on fasting glucose and insulin levels [11]. It should be noted that HOMA-IR reflects more hepatic than muscular insulin resistance [12]. Another marker of hepatic insulin resistance appears to be sex hormone-binding globulin (SHBG), which is produced mainly in the liver and synthesis of which is inhibited by hyperinsulinemia-compensating insulin resistance [13]. Low SHBG levels are associated with the severity of fatty liver, increased insulin levels, and HOMA-IR values [14]. Based on the knowledge that hepatic insulin resistance is related to excessive synthesis of glucose and triglycerides, the TyG index was recently proposed as a surrogate marker of liver steatosis and insulin resistance [15]. The limitation of all these markers in defining insulin resistance is the variability of the cut-off points for various populations and the moderate sensitivity and specificity of these cut-offs. The most widely used cut-off for HOMA-IR in the general population is 2.5 [16]. Notwithstanding, the European Group for the Study of Insulin Resistance (EGIR) suggests a lower cut-off point of 2.0 [17], and a similar value was established in Caucasian and Thai women with PCOS [18,19]. Recently, based on SHBG levels, we estimated the cut-off value for the HOMA-IR for young Caucasian women with PCOS to be 2.1 [20]. Similarly, the lack is a generally accepted cut-off point for SHBG, determining insulin resistance. In a study including 15,907 men and 42,034 women, the increased risk of type 2 diabetes development was observed at SHBG concentrations < 40 nmol/L and <50 nmol/L, respectively [21]. Recently, we estimated empirical optimal cut-off values for SHBG levels were ≤41.5 nmol/L, which is typical for insulin resistance in young Caucasian women with PCOS (area under ROC curve [AUC] 0.71, sensitivity [Se] 61.1%, specificity [Sp] 71.6%, positive predictive value [PPV] 70.7%, and negative predictive value [NPV] 62.1%) [22]. Also, the cut-off points estimated for the TyG index differ between studied populations. During 8.84 years of follow-up of the Spanish population, the TyG index value ≥ 8.31 was associated with an increased risk of type 2 diabetes development [23], while the cut-off points estimated in a population of young women with PCOS in the assessment of insulin resistance were 8.13 (Se 81.0%, Sp 68.0%) for Korean women, 8.51 (Se 63.2.0%, Sp 87.0%) for Chinese women, and 4.65 (Se 63.0%, Sp 60.0%) for Iranian women [24,25,26]. As was observed, the cut-off for TyG index values may be affected by age, race, and sex. There is a lack of studies estimating the cut-off value for the TyG index to discriminate the insulin resistance in a population of young Caucasian women with PCOS.

## 2. Materials and Methods

This study aimed to estimate the cut-off value for the TyG index discriminating insulin resistance based on the previously calculated HOMA-IR value and sex hormone-binding globulin (SHBG) level in women with polycystic ovary syndrome (PCOS) not exposed to any medicinal products.

We retrospectively analyzed medical records of 311 unselected Caucasian women for the first time who were diagnosed with PCOS based on the Rotterdam criteria [27] and admitted for routine complex endocrine and gynecologic diagnostics to the Department of Gynecological Endocrinology in 2019–2021.

Diagnosis of PCOS in women aged from 18 to 40 years old and a complete data set in the medical records are necessary for this analysis. The exclusion criteria for this analysis were other endocrinological disturbances, type 2 diabetes mellitus, arterial hypertension, and any pharmacological therapy including supportive treatment for obesity in the past and currently.

The analyzed data set included age, body mass, waist circumference, height, and routine measurements of fasting serum glucose, triglycerides, SHBG, and insulin. All assessments were performed in a single hospital laboratory using the same set of methods for all study subjects. Serum glucose and triglyceride concentrations were measured using the colorimetric method (Roche reagents Cobas c111, test number for glucose 4657527190 and triglycerides 04657594190). SHBG and insulin levels were determined using the ECLIA method (Roche Diagnostic GmbH, Mannheim, Germany, reagents for Cobas e411, test number for SHBG 750 and for insulin 650). Body mass index (BMI), HOMA-IR, and TyG index values were calculated with standard formulas:

HOMA-IR = fasting serum insulin level [µIU/mL) × fasting glucose level [mg/dL]/405.

TyG = ln [fasting triglyceride level (mg/dL) × fasting glucose level (mg/dL)/2].

As the retrospective analysis of patients’ records does not meet the criteria of a medical experiment, the approval of the Bioethical Committee was not required. All of the studied women gave consent for the standard procedure necessary to perform routine diagnostics only. The retrospective analysis includes anonymous patient data; thus, according to Polish law, additional consent was not required.

### 2.1. Data Analysis

During the analysis of medical records of 311 women with PCOS, it was found that 35 of them (11.2%) were diagnosed with thyroid diseases, 2 (0.6%) had type 1 and 5 (1.6%) had type 2 diabetes mellitus, and 8 (2.6%) had arterial hypertension. In accordance with the exclusion criteria, they were excluded from the analysis. Finally, 264 (94.9%) medical records were analyzed.

According to WHO criteria, normal weight was defined as body mass index values (BMI) from 18.5 to 24.9 kg/m^2^, overweight from 25.0 to 29.9 kg/m^2,^ and obesity as ≥30.0 kg/m^2^ [28]. Visceral obesity was diagnosed based on the International Diabetes Federation (IDF) as a waist circumference for Caucasian women ≥ 80 cm [29].

The cut-off point for HOMA-IR specific to insulin resistance was ≥2.1 [20]. The second cut-off for insulin resistance was a serum SHBG concentration ≤ 41.5 nmol/L [22].

### 2.2. Statistical Analysis

Statistical analysis was performed using STATISTICA 13.0 PL (TIBCO Software Inc., Palo Alto, CA, USA), StataSE 13.0 (StataCorp LLC, Lakeway Drive, TX, USA), and R software (R Core Team 2013). R: A language and environment for statistical computing. R Foundation for Statistical Computing, Vienna, Austria. URL http://www.R-project.org/) (accessed on 24 April 2024). Statistical significance was set at a *p*-value below 0.05. All tests were two-tailed. Imputations were not carried out for missing data. Nominal and ordinal data were expressed as numbers and percentages. Interval data were expressed as the mean ± standard deviation in the case of normal data distribution or as the median with lower and upper quartiles for non-normal data. The distribution of variables was evaluated by the W Shapiro–Wilk test and the quantile–quantile (Q-Q) plot. To find a cut-off point discriminating the insulin resistance based on the HOMA-IR value and SHBG level, parametric and non-parametric receiver–operating characteristic (ROC) curves were calculated with the area under the curve (AUC) and corresponding sensitivity (Se), specificity (Sp), positive and negative predictive values (PPV/NPV), with 95% confidence interval (CI), as well as with accuracy of classification (Acc). The ROC analysis was carried out with the ‘*cutpoint*’ and ‘*pROC*’ packages in R. To find an optimal, empirical cut-off point value for the TyG index, the Youden J statistic (index) was used, which assesses the performance of a diagnostic test. It was assessed as sensitivity + specificity − 1. Moreover, in the analysis of the cut-off point, the maximization of the Youden index parametrically, assuming normally distributed data in both classes, was used.

## 3. Results

### 3.1. Study Group

The characteristics of the studied non-medicated women with PCOS are listed in Table 1. Concordantly with WHO criteria, overweight was diagnosed in about one-quarter of study subjects and obesity in 23.4%. Based on the waist circumference cut-off point for Caucasian women, visceral obesity was found in more than half of the analyzed women. Hypertriglyceridemia was a rare metabolic disturbance, observed in 9.5%, whereas impaired fasting glucose was much more frequent, at 59.5%. The values of HOMA-IR ≥ 2.1 were found in 37.1%. Finally, SHBG levels ≤ 41.5 nmol/L were present in 46.2% of the studied women.

### 3.2. The Cut-Off Points for the TyG Index for Insulin Resistance Assessment Based on HOMA-IR Values and SHBG Levels

**Figure 1:**
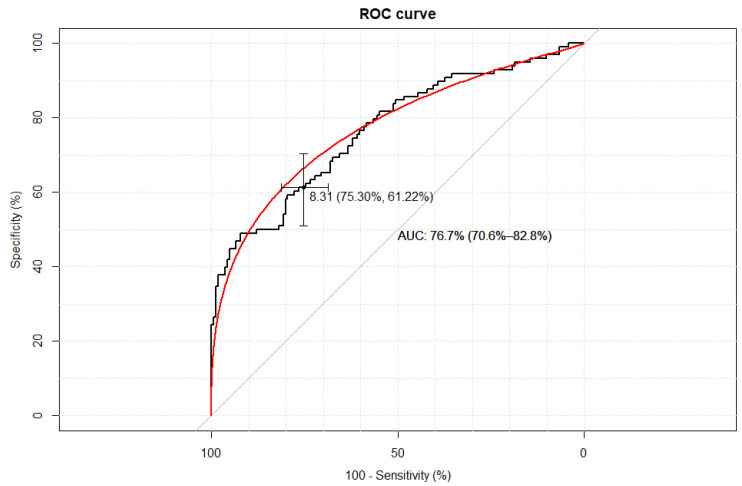
The receiver–operating characteristic curve (ROC curve) of the TyG index value for the assessment of insulin resistance based on HOMA-IR value ≥ 2.1. The figure presents a calculation of the area under curve (AUC) with a 95% confidence interval (CI).

The empirical optimal cut-off values for the TyG index corresponding to HOMA-IR values ≥ 2.1 were ≥8.31 AUC 0.77, Accuracy 0.70, Se 61.2% (95% CI: 50.8–70.7%), Sp 75.3% (95% CI: 67.9–81.5%), PPV 59.4% (95% CI: 49.2–68.9%), and NPV 76.7% (95% CI: 69.3–82.8%)—Figure 1.

**Figure 2:**
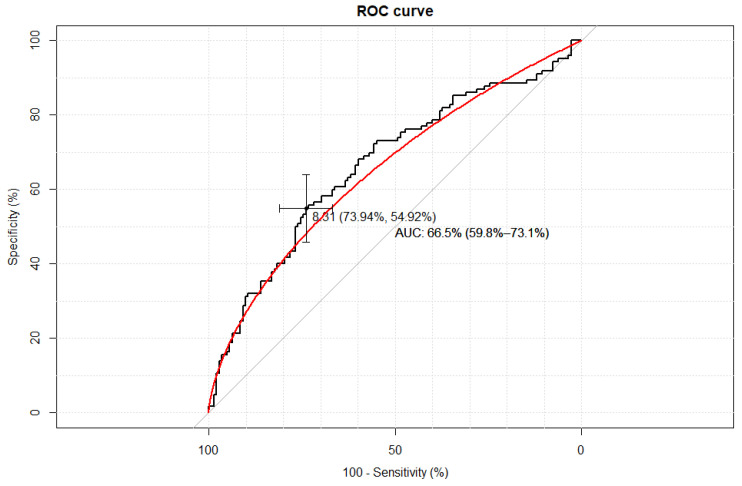
The receiver–operating characteristic curve (ROC curve) of the TyG index value for assessment of insulin resistance based on SHBG concentration ≤ 41.5 nmol/L. The figure presents a calculation of the area under curve (AUC) with a 95% confidence interval (CI).

Empirical optimal cut-off values for the TyG index corresponding to SHBG levels ≤ 41.5 nmol/L were ≥8.31 AUC 0.67, Accuracy 0.65, Se 54.9% (95% CI: 45.7–63.8%), Sp 73.9% (95% CI: 65.8–80.8%), PPV 64.4% (95% CI: 35.6–45.6%), and NPV 65.6% (95% CI: 57.7–72.8%)—Figure 2.

Of note, the same cut-off values for the TyG index were established in both methods, but with a greater AUC for HOMA-IR. Comparing both methods, the PPV value was higher for cut-off values based on the SHBG level, while the NPV value was higher for cut-off values based on the HOMA-IR value. Moreover, no significant difference was observed for specificity, while the sensitivity was higher for cut-off values based on the HOMA-IR value rather than on the SHBG level.

### 3.3. Probability of Insulin Resistance Assessed Based on HOMA-IR Values and SHBG Levels According to the TyG Index Values

**Figure 3:**
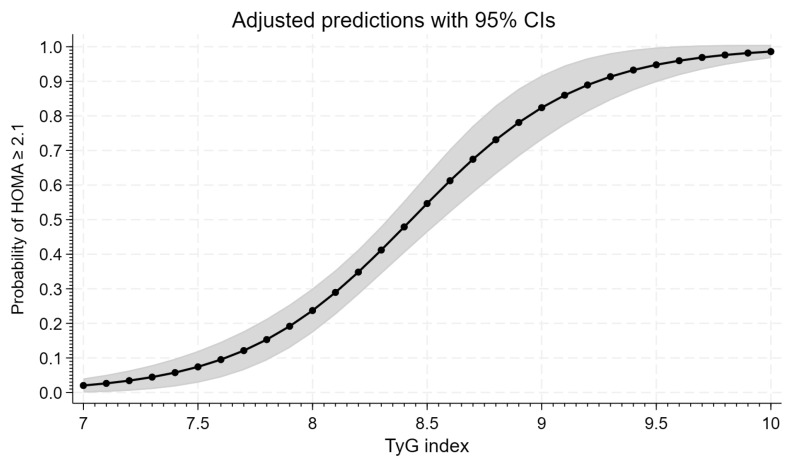
Logistic regression curve for the TyG index and probability of HOMA-IR value ≥ 2.1. Gray zone shows 95% confidence interval (CI).

Figure 3 graphically presents the course of the logistic regression model curves with a 95% confidence interval, illustrating the probability of insulin resistance (HOMA-IR ≥ 2.1) for the analyzed TyG index. The 50% probability of the HOMA-IR value ≥ 2.1 is obtained for a TyG index value ≥ 8.431.

**Figure 4:**
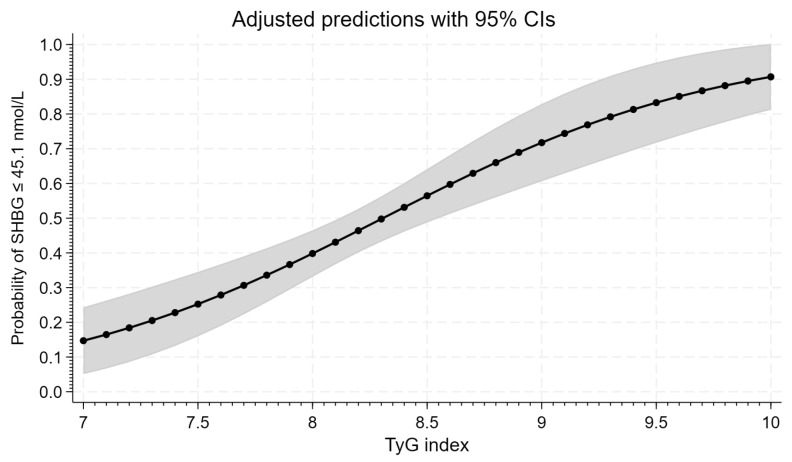
Logistic regression curve for the TyG index and probability of SHBG level ≤ 41.5 nmol/L. Gray zone shows 95% confidence interval (CI).

Figure 4 graphically presents the course of logistic regression model curves with a 95% confidence interval, illustrating the probability of a SHBG value ≤ 41.5 nmol/L for the analyzed TyG index. The 50% probability of a SHBG value ≤ 41.5 nmol/L is obtained for a TyG index value ≥ 8.307. It is worth noting that the obtained values are close to those obtained in the ROC analysis, considering the variability range of the given indicator.

## 4. Discussion

This is the first study that estimates the triglyceride–glucose index (TyG) cut-off value, discriminating insulin resistance based on the HOMA-IR value and SHBG level in young Caucasian women with PCOS.

The empirically estimated HOMA-IR cut-off point for Caucasian women with PCOS was ≥2.1 [20], and for a SHBG level ≤ 41.5 nmol/L [22]. Based on these cut-off points, we estimated the cut-off point for the TyG index—8.31 (sensitivity 61.2%, specificity 75.3%, and sensitivity 54.9%, specificity 73.9%), which is lower than that assumed for the general population—8.55 [30], and for Asian women with PCOS—8.51 [25]. This cut-off point is also lower than that estimated in the Korean population, where the cut-offs for predicting the incidence of metabolic syndrome were 8.72 for the TyG index and 1.8 for HOMA-IR. The cut-off points for predicting incident metabolic syndrome were 8.52 and 1.5, respectively [31]. However, it should be noted that it is the same as that estimated in the Spanish population (4.820 patients from the Vascular–Metabolic CNS study cohort) to determine the risk of developing type 2 diabetes [23]. Thus, despite the relatively low sensitivity and moderate specificity, the cut-off point for a TyG index ≥ 8.31 should be recommended for use in both studies and clinical practice to determine the risk of insulin resistance. It should also be noted that this sensitivity and specificity were slightly lower than that obtained in the population of Chinese women with PCOS (Se: 61.2% and 54.9% vs. 63.2% and Sp: 75.3% and 73.0% vs. 87.0%, respectively) [25]. It should also be mentioned that the TyG index cut-off point estimated in our study was higher than in the population of 14,498 NHANES participants of reproductive age (8.31 vs. 8.12). The sensitivity of the TyG index cut-off point estimated in our study was slightly lower than in the analysis of the NHANES data (61.2% and 54.9% vs. 63.0%), but the specificity was slightly higher (75.3% and 73.0% vs. 70%). This difference may be due to racial variability. The NHANES population included women of various races. Interestingly, the HOMA-IR cut-off point used in the analysis of the NHANES data was similar to that used in our study (2.2 vs. 2.1) [32]. It should also be noted that, despite the use of two methods to establish the cut-off point for the TyG index in our study, the value was the same. However, a greater AUC was obtained for HOMA-IR-based analysis. The PPV value was higher for the cut-off established based on the SHBG level, while the NPV value was higher for the cut-off based on the HOMA-IR value. The specificity was similar and the sensitivity was higher for cut-off values based on HOMA-IR rather than SHBG. These results, in terms of obtaining the same cut-off point value for the TyG index using different methods, confirm its diagnostic value.

In summary, regardless of the described caveats, the results of our study indicate that the TyG index can be used as a marker of insulin resistance in women with PCOS. Their use may help reduce the cost of diagnostics because they do not require more expensive insulin testing. Additionally, the results of our study confirm the need to verify the cut-off points of insulin resistance indices in women with PCOS, considering race, and that the cut-off points established for the general population cannot be extrapolated to the population of women with PCOS.

We also analyzed the probability of insulin resistance assessed on the basis of HOMA-IR values and SHBG levels according to the TyG index using the logistic regression model curves with a 95% confidential interval. This analysis showed that a 50% probability of a HOMA-IR value ≥ 2.1 is obtained for a TyG index value ≥ 8.431. Thus, these TyG index values were slightly higher than those established in our population in the receiver–operating characteristic curve based on both HOMA-IR values and SHBG levels (8.31), while a 50% probability of a SHBG value ≤ 41.5 nmol/L is obtained for the TyG index value ≥ 8.307. It is worth noting that these TyG index values were the same as established in our population in the ROC curve analysis. This indicates that after determining the risk of insulin resistance based on the TyG index, insulin resistance should be confirmed using HOMA-IR or SHBG, eventually.

### 4.1. The Strength of This Study

The strength of our study is that it comprises a homogeneous study group of non-medicated Caucasian women with diagnosed PCOS with different nutritional statuses. It should be emphasized again that this is the first study of this type conducted in a population of European Caucasian women diagnosed with PCOS. Moreover, in previous studies, the cut-off points of the TyG index both in the population of women with PCOS and in the general population were determined based on HOMA-IR values only, whereas in our analysis, the second surrogate marker of hepatic insulin resistance, i.e., serum SHBG concentrations was used for this purpose.

### 4.2. The Limitations of This Study

This single-center study undoubtedly has some limitations, the most important of which include this study’s retrospective nature and the use of biochemical tests performed at different times as part of routine clinical diagnostics. The collection of biological material and simultaneous performance of tests using the same series of reagents decreased the variability of glucose, triglycerides, SHBG, and especially insulin concentrations, which could have occurred when using different reagent series. However, it seems that this did not have a significant impact on the obtained results. Another limitation of this study was that only Caucasians were included and there was a lack of a control group of women without a diagnosis of PCOS. This would have allowed us to determine whether the results obtained in our study could be extrapolated to the general population of young women. Additionally, for the estimation of the cut-off point value for the TyG index, we used surrogate markers of insulin resistance, not HIEC. However, it should be noted that the surrogate markers used in our study reflect more liver than muscle insulin resistance, similar to the TyG index.

## 5. Conclusions

Our study suggests that the cut-off point for the TyG index, which discriminates insulin resistance based on both HOMA-IR and SHBG values, in young Caucasian women with PCOS, is 8.31. In addition, our data confirm the usefulness of the TyG index as an initial assessment of insulin resistance, which should be confirmed by assessing the HOMA-IR value or SHBG concentration.

## Figures and Tables

**Table 1:** The characteristics of the studied non-medicated women with PCOS.

	Study Group N = 264
Age [years]	26 ± 5
Body mass [kg]	68.0 (59.0; 83.9)
BMI [kg/m^2^]	24.8 (21.3; 29.4)
Overweight [N-%]	68–25.8
Obesity [N-%]	62–23.4
Waist circumference [cm]	84.9 ± 15.9
Visceral obesity [N-%]	144–54.6
Triglicerides [mg/dL]	95.3 ± 49.0
Hypertrigliceridemia [N-%]	25–9.5
Glucose [mg/dL]	84.9 ± 5.8
Impaired fasting glucose [N-%]	157–59.5
Insulin [uIU/mL]	7.9 (5.6; 12.0)
HOMA-IR	2.2 ± 2.0
HOMA-IR ≥ 2.1 [N-%]	98–37.1
SHBG [nmol/L]	46.4 (30.2; 64.8)
SHBG ≤ 41.5 [nmol/L] [N-%]	122–46.2
TyG index	8.16 (7.87; 8.46)

Mean ± standard deviation (for variables with normal distribution) or median with lower and upper quartile in brackets (for variables with non-normal distribution).

## Data Availability

Data are available from the corresponding author.

## Abbreviations

The following abbreviations are used in this manuscript:

AUC	area under curve
BMI	body mass index
EGIR	European Group for the Study of Insulin Resistance
HIEC	hyperinsulinemic–euglicemic
HOMA-IR	homeostatic model assessment insulin resistance
NHANES	National Health and Nutrition Examination Surveys
NPV	negative predictive value
PCOS	polycystic ovary syndrome
PPV	positive predictive value
Se	sensitivity
SHBG	sex hormone-binding globulin
Sp	specificity
TyG	triglyceride–glucose index

## References

[1] Gilling-Smith C., Willis D.S., Beard R.W., Franks S. (1994). Hypersecretion of androstendione by isolated thecal cells from polycystic ovaries. J. Clin. Endocrinol. Metab..

[2] Zhu S., Sun F., Li W., Cao Y., Wang C., Wang Y., Liang D., Zhang R., Zhang S., Wang H. (2011). Apelin stimulates glucose uptake through the PI3K/Akt pathway and improves insulin resistance in 3T3-L1 adipocytes. Mol. Cell. Biochem..

[3] Moran L.J., Misso M.L., Wild R.A., Norman R.J. (2010). Impaired glucose tolerance, type 2 diabetes and metabolic syndrome in polycystic ovary syndrome: A systematic review and meta-analysis. Hum. Reprod. Update.

[4] Rizzo M., Berneis K. (2005). Lipid triad or atherogenic lipoprotein phenotype: A role in cardiovascular prevention?. J. Atheroscler. Thromb..

[5] Wild R.A. (2002). Long-term health consequences of PCOS. Hum. Reprod. Update.

[6] Guastella E., Longo R.A., Carmina E. (2010). Clinical and endocrine characteristics of the main polycystic ovary syndrome phnotypes. Fertil. Steril..

[7] Wiltgen D., Spritzer P.M. (2010). Variation in metabolic and cardiovascular risk in women with different polycystic ovary syndrome phenotypes. Fertil. Steril..

[8] Chittenden B.G., Fullerton G., Maheshwari A., Bhattacharya S. (2009). Polycystic ovary syndrome and the risk of gynecological cancer: A systematic review. Reprod. Biomed. Online.

[9] Jakimiuk A.J., Issat T. (2009). PCOS and cancer risk. Folia Histochem. Cytobiol..

[10] Wallace T.M., Levy J.C., Matthews D.R. (2004). Use and abuse of HOMA modeling. Diabetes Care.

[11] Bonora E., Targher G., Alberiche M., Bonadonna R.C., Saggiani F., Zenere M.B., Monauni T., Muggeo M. (2000). Homeostasis model assessment closely mirrors the glucose clamp technique in the assessment of insulin sensitivity: Studies in subjects with various degrees of glucose tolerance and insulin sensitivity. Diabetes Care.

[12] Tripathy D., Almgren P., Tuomi T., Groop L. (2004). Contribution of insulin-stimulated glucose uptake and basal hepatic insulin sensitivity to surrogate measures of insulin sensitivity. Diabetes Care.

[13] Akin F., Bastemir M., Alkiş E., Kaptanoglu B. (2009). SHBG levels correlate with insulin resistance in postmenopausal women. Eur. J. Intern. Med..

[14] Ye J., Yao Z., Tan A., Chen Y., Li X., He R., Tang R., Hu Y., Zhang H., Yang X. (2017). Low serum sex hormone-binding globulin associated with insulin resistance in men with nonalcoholic fatty liver disease. Horm. Metab. Res..

[15] Simental-Mendía L.E., Rodríguez-Morán M., Guerrero-Romero F. (2008). The product of fasting glucose and triglycerides as surrogate for identifying insulin resistance in apparently healthy subjects. Metab. Syndr. Relat. Disord..

[16] Matthews D.R., Hosker J.P., Rudenski A.S., Naylor D.R., Treacher D.F., Turner R.C. (1985). Homeostasis model assessment: Insulin resistance and beta-cell function from fasting plasma glucose and insulin concentrations in man. Diabetologia.

[17] Balkau B., Charles M.A. (1999). Comment on the provisional report from the WHO consultation. European Group for the Study of Insulin Resistance (EGIR). Diabet. Med..

[18] Jensterle M., Weber M., Pfeifer M., Prezelj J., Pfutzner A., Janez A. (2008). Assessment of insulin resistance in young women with polycystic ovary syndrome. Int. J. Gynaecol. Obstet..

[19] Wongwananuruk T., Rattanachaiyanont M., Leerasiri P., Indhavivadhana S., Techatraisak K., Angsuwathana S., Tanmahasamut P., Dangrat C. (2012). The usefulness of Homeostatic Measurement Assessment-Insulin Resistance (HOMA-IR) for detection of glucose intolerance in Thai women of reproductive age with Polycystic Ovary Syndrome. Int. J. Endocrinol..

[20] Biernacka-Bartnik A., Kocełak P., Owczarek A.J., Choręza P., Markuszewski L., Madej P., Pudzianowska-Kuźnicka M., Chudek J., Olszanecka-Glinianowicz M. (2023). The cut-off value for HOMA-IR discriminating the insulin resistance based on the SHBG level in women with polycystic ovary syndrome. Front. Med..

[21] O’Reilly M.W., Glisic M., Kumarendran B., Subramanian A., Manolopoulos K.N., Tahrani A.A., Keerthy D., Muka T., Toulis K.A., Hanif W. (2019). Serum testosterone, sex hormone-binding globulin and sex-specific risk of incident type 2 diabetes in a retrospective primary care cohort. Clin. Endocrinol..

[22] Biernacka-Bartnik A., Kocełak P., Owczarek A.J., Choręza P., Markuszewski L., Madej P., Pudzianowska-Kuźnicka M., Chudek J., Olszanecka-Glinianowicz M. (2022). Prediction of insulin resistance and impaired fasting glucose based on sex hormone-binding globulin (SHBG) levels in polycystic ovary syndrome. Int. J. Endocrinol..

[23] Navarro-Gonzalez D., Sanchez-Inigo L., Pastrana-Delgado J., Fernandez-Montero A., Martinez J.A. (2016). Triglyceride-glucose index (TyG index) in comparison with fasting plasma glucose improved diabetes prediction in patients with normal fasting glucose: The Vascular-Metabolic CUN cohort. Prev. Med..

[24] Kwon S., Heo A., Chun S. (2023). Triglyceride and glucose index for identifying abnormal insulin sensitivity in women with polycystic ovary syndrome. Obstet. Gynecol. Sci..

[25] Zheng Y., Yin G., Chen F., Lin L., Chen Y. (2022). Evaluation of Triglyceride Glucose Index and Homeostasis Model of Insulin Resistance in Patients with Polycystic Ovary Syndrome. Int. J. Womens Health.

[26] Kheirollahi A., Teimouri M., Karimi M., Vatannejad A., Moradi N., Borumandnia N., Sadeghi A. (2020). Evaluation of lipid ratios and triglyceride-glucose index as risk markers of insulin resistance in Iranian polycystic ovary syndrome women. Lipids Health Dis..

[27] Thessaloniki ESHRE/ASRM-Sponsored PCOS Consensus Workshop Group (2008). Consensus on infertility treatment related to polycystic ovary syndrome. Hum. Reprod..

[28] Hosseini S.M. (2017). Trigliceride–Glucose Index Simulation. J. Clin. Basic Res..

[29] WHO (1998). Obesity: Preventing and Managing the Global Epidemic.

[30] The IDF Consensus Worldwide Definition of the Metabolic Syndrome. www.idf.org/webdata/docs/MetS_def_update2006.pdf.

[31] Son D.-H., Lee H.S., Lee Y.-J., Lee J.-H., Han J.-H. (2022). Comparison of triglyceride-glucose index and HOMA-IR for predicting prevalence and incidence of metabolic syndrome. Nutr. Metab. Cardiovasc. Dis..

[32] Xia W., Cai Y., Zhang S., Wu S. (2023). Association between different insulin resistance surrogates and infertility in reproductive- aged females. BMC Public Health.

